# Annotations

**Published:** 1894-11-10

**Authors:** 


### ANNOTATIONS.

### Tuberculosis in Cattle.

A DISCUSSION is going on among veterinarians as to whether tuberculosis is common or rare among young cattle, cattle, that is to say, of the age of six to eighteen months. Some maintain that the disease is comparatively common, whilst others declare that even if it be so, no great harm is done, inasmuch as the disease may never get beyond the latent stage. However the actual facts may be, this at any rate is quite certain, that the communicability of tuberculosis is a subject about which real knowledge is still quite in its infancy, even in regard to human medicine; much more, therefore, is this the case in regard to veterinary practice. If animals affected with tuberculosis can be fattened and made fit for the table, are the bacilli in their flesh or internal organs destroyed by cooking? Is the milk of animals infected by tuberculosis which is quiescent, injurious to those who drink it? All these are questions of the very first order of practical importance. They are questions which cannot now be authoritatively answered either by physicians or veterinarians, and it is foolish and unscientific in the last degree to attempt to dogmatise about them in the present state of our knowledge. It is to be hoped, however, that the long expected report of the Commission on Tubercolosis will, when it is issued, throw some light upon these very important problems.

### The Public and the London Medical Schools.

THE *Times* has opened its columns to a correspondence on "the public neglect of medical education in London," and in so doing has rendered a conspicuous service to the medical profession. The medical schools of the metropolis are second to none in the world, and yet it is remarkable that with few and unimportant exceptions they may be spoken of as private ventures, which are without private and State endowment, and carry on their work without help from outside. At the time they were founded, the "plant" of a medical school was very little, and the cost of maintenance quite small, and the one great requisite was a body of capable and energetic teachers. With the enormous development of science in its application to medicine within recent years this has greatly changed. The plant now required is very costly, and the maintenance of efficient laboratories is a very heavy expense. There is every prospect that this expense will increase. To the chemical laboratory has been already added the physiological laboratory, and to that must soon be added a bacteriological laboratory, where the methods of modern pathological research can be taught, and its great results familiarised to the student. It has been generally admitted that education is not to be conducted on self-supporting lines. In our primary schools we give free education, and the State pays the cost. Universities are richly endowed, and are continually attracting large benefactions. But medical schools which are imparting a highly technical and costly education, and in whose proper conduct and equipment the public are more directly interested than in any other schools, are in London practically unendowed. It is not difficult to point to the cause of this apparent anomaly. It is to be found, in the first place, in the private nature of these schools. They have been started by individuals, managed by the teachers only, and "run" for private profit; indeed, there are very few institutions whose accounts are more jealously kept secret than are those of medical schools. The absence of an University in which the schools should be constituent colleges has emphasised their "private" nature. The result of the absence of endowment is equally apparent. To pay their way expenses often have to be cut down, and unless a school is attracting a large number of students the temptation to starve its most expensive departments is very strong. If the present unsatisfactory and unfair state of affairs is to be remedied, the medical schools must cease to be private ventures, and must be managed by committees or councils, on which laymen will have a certain voice. When this change in their constitution is effected, and there is a concentration of the teaching on all except the strictly medical subjects in a smaller number of schools, we cannot doubt that the public will come forward to support the London medical schools, and to relieve the teachers of a burden which ought not to press on their shoulders alone.

### Infantile Scurvy.

THE Bradshaw Lecture, delivered at the College of Physicians on Tuesday by Dr. T. Barlow, was full of good matter, interesting not only to medical men but to that large class of modern mothers who persist in bringing up their infants on all kinds of artificial substitutes for their proper and natural food. While in obedience to the dictates of selfishness and fashion the mother's breast is year by year being less used, and except as a foundation for dress is becoming a useless and meaningless appendage, infantile diseases are increasing and apparently taking new forms. We have long known of rickets as a common result of improper feeding, but of late years, combined with that disease, scurvy has been observed. So marked and striking are both the symptoms and the pathological appearances that, as Dr. Barlow justly enough says, they could hardly have escaped the observation of men like Sir W. Jenner, who gave such admirable descriptions of rickets, if they had existed in those days, and the conclusion is unavoidable that this complication is a new nemesis of our too complex civilisation, a new punishment for our revolt against natural laws. The symptoms of infantile scurvy are apt to come on somewhat suddenly, and most commonly between the ninth and eighteenth month. They take the form of swelling and pain, with extreme sensitiveness to movement, of the lower limbs; some œdema of the feet, weakness of the back, great pallor, often crepitus from fracture through the junction of the epiphyses with the shaft of the bone; sometimes an extraordinary sinking backwards of the sternum from fracture of sternal ends of ribs; proptosis from effusion behind the eyeball, and where there are teeth spongy gums It should be remembered, however, that while in some cases the symptoms of the accompanying rickets may be clinically unrecognisable, in others the scurvy may show itself by nothing more than pallor, irritability and greater tenderness than usual. In these cases, however, the test is treatment by antiscorbutic food, a few days often making a surprising difference. First among the causes is the use of so-called patent foods, especially those for the preparation of which very little milk is used; next comes the use of condensed milk; and last, the use of over-diluted milk. It is an interesting thing to hear that while rickets is abundantly prevalent among the children of the poor, scurvy rather belongs to the well-to-do, the reason probably being that the poor cannot afford the patent foods, and especially that their children feed with the parents and thus obtain potatoes at an early age. The treatment is fresh milk, raw meat, juice, freshly cooked sieved potatoes, juice of grapes, oranges, and other fruits. No treatment is of any avail without antiscorbutics, and they are effectual directly. Dr. Barlow throws doubts even on milk when it has been subjected to prolonged boiling.

